# Laryngeal sensory neuropathy caused by COVID-19: findings using laryngeal electromyography

**DOI:** 10.1007/s00405-023-07895-0

**Published:** 2023-03-17

**Authors:** Paulina Krasnodębska, Agata Szkiełkowska, Beata Miaśkiewicz

**Affiliations:** Institute of Physiology and Pathology of Hearing, Warsaw, Poland

**Keywords:** Laryngeal neuropathy, COVID-19, SARS-CoV-2, Laryngeal electromyography (LEMG), Voice

## Abstract

**Purpose:**

Laryngeal sensory neuropathy (LSN) is caused by a disorder of the superior laryngeal nerve or the recurrent laryngeal nerve. A diagnosis of LSN should include laryngeal electromyography (LEMG) and laryngovideostroboscopy (LVS). The aim of this study was to characterize the physical and subjective symptoms of neuropathy in patients diagnosed with LSN following COVID-19.

**Material and methods:**

Since the beginning of the COVID-19 pandemic, 6 patients who had recovered from the disease presented to us with LSN symptoms. All patients underwent laryngological and phoniatric examination, objective and subjective voice assessment, and LEMG.

**Results:**

The most common LSN symptom reported by patients was periodic hoarseness of varying severity. Other common symptoms were the sensation of a foreign body in the throat and voice fatigue. Endoscopy often showed functional abnormalities. The LSN patients could be characterized by LEMG recordings, and all showed abnormal activity of the cricothyroid (CT) muscle. The degree of EMG changes in the CT correlated moderately with the severity of dysphonia.

**Conclusions:**

Sensory neuropathy of the larynx may be a long-lasting complication of SARS-COV-2 infection. The severity of EMG neuropathic changes in the CT muscle broadly corresponds to the severity of dysphonia.

## Introduction

Sensory neuropathies appear under a diverse array of conditions. The best known examples are diabetic peripheral neuropathy and trigeminal neuralgia. The pathogenesis results from nerve degeneration caused by metabolic damage, mechanical trauma, or viral infection [1]. Laryngeal sensory neuropathy (LSN) is caused by disorders of the superior laryngeal nerve or the recurrent laryngeal nerve. Symptoms of the disease may be throat discomfort (ranging from paresthesia to numbness, often including pain), permanent dysphonia, laryngospasm, or chronic cough [2–6]. Diagnosis of LSN should include laryngeal electromyography (LEMG) and laryngovideostroboscopy (LVS) [7].

In differential diagnosis several well-defined clinical syndromes have overlapping symptomatology: laryngo-pharyngeal reflux, postnasal drip, chronic refractory cough, globus pharyngeus, paradoxical vocal fold movement, and muscle tension dysphonia [8]. It is hypothesized that in all these states laryngeal hypersensitivity is a common underlying factor [9]. In cases of COVID-19 infection, neuropathy may be caused by viral mechanisms and inflammatory responses (host cytokines and release of chemokines) [10–12]. Nervous system disorders are present in 36% of individuals infected with SARS-CoV-2 [13, 14].

In the literature, laryngeal paralysis arising from innervation damage is the most common complication due to COVID-19 [15]. Sensory neuropathy is seldom mentioned as a complication, mentioned only as co-existing with movement dysfunction [16, 17]. When a search was done of medical databases, using the keywords "COVID" or "SARS" and "laryngeal sensory neuropathy" without time limit, we found only 1 article in PubMed and 54 in Google Scholar.

## Aim

The aim of this study was to examine patients who came to the Audiology and Phoniatrics Clinic, after suffering SARS-CoV-2 infection and having LSN as a complication. We wanted to characterize the physical and subjective manifestations of the neuropathy, including any electromyographic features.

## Material

The material of the study consisted of patients presenting to our hospital with symptoms suggestive of post COVID-19 sensorineural dysfunction of the larynx. The patients were diagnosed with LSN and referred for LEMG testing. Since the beginning of the pandemic, 6 patients presenting with LSN symptoms came under our care. All of them had had COVID-19 infection confirmed by a SARS-COV-2 RNA test. There was 1 male and 5 females, with a mean age of 50.6 years (SD 10.9). None of the 6 patients had been in a critical state or required admission to an intensive care unit due to COVID-19. The inclusion criteria for the study were the duration of symptoms, the acute onset of laryngeal-related sensory symptoms and ruling out any other cause of the disorder. Patients were included in the study only when other previously undiagnosed common etiologies for throat discomfort had been excluded, based mainly on previous neurological, pulmonological, or cardiological diagnosis. Other etiologies considered in the differential diagnosis were: cricopharyngeal dysfunction, recent upper respiratory tract infection, current smoking, untreated asthma, untreated rhinitis, untreated gastroesophageal reflux, significant psychological factors (such as psychosis, schizophrenia, or mood disorders that prevented participation in the assessment), or neurological impairment [9]. During diagnostic hospitalisation at our clinic, in addition to the LEMG examination, patients underwent phoniatric and speech therapy assessment to exclude other laryngeal diseases such as muscle tension dysphonias. We have excluded one patient from the statistical analysis (Patient 6). Due to a history of previous right vocal fold dysfunction, electromyographic recordings from the muscles on the right side were not included in further analysis.

In all patients the symptoms that were the reason for referral for otolaryngologic and phoniatry diagnosis appeared up to 1 month after COVID-19 and lasted a minimum of 3 months, which meets the criteria for post-viral vagal neuropathy (PVVN) as a result of COVID-19 [17–19]. The general characteristics of the patients are listed in Table 1. Detailed descriptions of the patients' cases are presented below.

**Table 1:** Details of 6 patients suffering laryngeal sensory neuropathy (LSN) and their symptoms

Patient no	Age	Sex	Date of COVID-19 infection	Date of phoniatric examination and EMG	Symptoms
1	59	F	11.2020	3.2022	Vocal fatigue
					Voice fading
					Periodic hoarseness
2	43	F	4.2021	2.2022	Vocal fatigue
					Periodic hoarseness
					Dry throat
3	44	F	5.2021	1.2022	Periodic hoarseness
					Painless sensation of a foreign body in the throat
4	40	F	11.2021	6.2022	Painful sensation of a foreign body in the throat
					Sensation of tightening of the larynx
5	47	F	11.2021	6.2022	Vocal fatigue
					Painful sensation of a foreign body in the throat (right side)
					Periodic hoarseness
					Dry throat
6	71	M	3.2021	11.2021	Vocal fatigue
					Periodic hoarseness
					Painless sensation of a foreign body in the throat
					Sensation of tightening in the larynx

## Description of patients

### Patient 1

A 59 year old female suffering from asthma, hypothyroidism, and arterial hypertension was referred for a phoniatric diagnosis due to a 1-year history of hoarseness, vocal fatigue, and voice weakness. The complaints started about a month after COVID-19. In addition to vocal complaints, the patient developed an exacerbation of asthma requiring intensification of pulmonary treatment. At the time of admission to the phoniatry department, the patient’s asthma had been stabilized.

### Patient 2

A 43 year old female voice worker, without comorbidities, was referred for a phoniatric evaluation due to laryngeal dysfunction after COVID-19 infection. The complaints had lasted 10 months. Initially, intercostal nerve paralysis, paradoxical vocal folds movement, right-sided trigeminal neuralgia, and muscle weakness on the right side of the body were diagnosed. In addition to periodic hoarseness, the patient complained of voice fatigue while speaking, dry throat, and a problem with breath coordination during vocalization. The patient had had a neurological diagnosis prior to admission to the phoniatry department, which excluded other neurological causes of the symptoms.

### Patient 3

A 44 year old female teacher, with a history of seasonal allergy, was referred for a phoniatric evaluation due to laryngeal dysfunction after COVID-19 infection. The complaints had lasted for 8 months. Initially, she complained of hoarseness, increased dryness in the throat, impaired concentration and attention, and general irritability. The patient reported a sensory disturbance on the scalp and in the nasal area for a couple of months after the infection. The patient had a gastrological, otolaryngological, neurological, and rheumatological evaluation prior to admission to the phoniatry department, ruling out other causes of the laryngeal dysfunction.

### Patient 4

A 40 year old female voice worker, without comorbidities, was referred for a phoniatric evaluation due to laryngeal dysfunction after COVID-19 infection. The complaints had lasted for 7 months. The patient initially reported choking while eating. In addition, she had persistent sore throat (aggravated after speaking) and feelings of tightness and burning in the throat. She did not complain of hoarseness. Gastrointestinal dysfunction had been ruled out before admission to hospital.

### Patient 5

A 47 year old female with a 2-year history of trigeminal neuralgia was referred for phoniatric evaluation due to right-sided pain in the throat, dry throat, and voice fading following COVID-19 infection 8 months earlier. A neurological examination showed no abnormalities. Due to a previous history of neuralgia, the neurologist decided to administer pregabalin.

### Patient 6

A 71 year old male with chronic heart failure and a 6-year history of right-sided laryngeal paralysis after thyroid surgery (laryngeal laryngoplasty with hyaluronic acid) was referred for a phoniatric evaluation due to laryngeal dysfunction exacerbation after COVID-19 infection. After the infection, the patient noticed a feeling of discomfort in the larynx, increased fatigue of the voice, and memory deterioration. The patient was reviewed by a pulmonologist, cardiologist, and neurologist. He did not require modification of treatment due to the absence of changes in the medical examinations. Due to a history of previous right vocal fold dysfunction, electromyographic recordings from the muscles on the right side were not included for further analysis.

### Methods

Procedures consisted of a laryngological and phoniatric examination, objective and subjective voice assessment, and LEMG. Endoscopy of the larynx was performed through the nose in two ways: using a flexible 3.2 mm diameter Xion fiberoptic scope and a rigid endoscope. Evaluation with a flexible endoscope avoids misclassification of hyperfunctional features due to a forced tongue position during examination with rigid optics. Laryngovideostroboscopy (LVS) was done and classified according to standards in the literature [20–22]. The following stroboscopic parameters were evaluated: amplitude, mucosal waveform, closed phase, phase differences, and symmetry of vibration. Acoustic voice analysis was assessed according to the Yanagihara scale, assigning a grade of hoarseness on the basis of the spectrogram. Perceptual voice evaluation was based on the 4-point GRBAS scale (G, grade; R, roughness; B, breathiness; A, asthenicity; S, strain) [23]. All patients also completed a detailed self-assessment with the Voice Handicap Index (VHI) questionnaire.

For LEMG evaluation we used the standard percutaneous approach with a concentric needle electrode used clinically for diagnosis of the thyroarytenoid (TA) and cricothyroid (CT) muscles [24]. The procedure records electrical responses of branches of the vagus nerve supplying motor and sensory innervation to the larynx via the recurrent laryngeal nerve and the superior laryngeal nerve. Accurate electrode placement was confirmed by asking the patient to phonate a sustained /a/ and /e/ and seeing appropriate neural activity. The ground electrode was placed at the midline over the forehead [25]. Recordings were made using the Neurosoft EMG apparatus.

All procedures were approved by our bioethics committee (KB.IFPS 1/2021). Relationships between laryngeal parameters in patients with LSN were assessed using Spearman correlations.

## Results

Since the beginning of the COVID-19 outbreak, six patients with symptoms fulfilling the criteria for the diagnosis of laryngeal sensory neuropathy due to SARS-COV-2 infection have been referred to our clinic. As shown in Table 1, these patients presented mainly within the last year. This was due to epidemiological strictures and limited clinical activity during the outbreak. Table 1 shows the epidemiological and interview data. Half the subjects were professional voice users. The most common LSN symptom reported by patients was periodic hoarseness of varying severity ( in 83% of them). Other frequently reported symptoms were the sensation of a foreign body in the throat (two-thirds of them), voice fatigue (two-thirds), dry throat (one-third), and a sensation of tightness in the throat (one-third).

Table 2 shows the results of LVS examination and voice assessment. On endoscopic examination, functional abnormalities in the form of hyperfunctional dysphonia were mainly observed. Distinctive features of hyperfunctional dysphonia observed by LVS were: supraglottic hypertension, reduced open phase, reduced maximum amplitude, and elongated closed phase [21]. We observed these abnormalities in two-thirds of our patients. In 2 of the first 5 patients we observed periodic abnormal movements of the vocal folds. Acoustic voice analysis confirmed varying degrees of hoarseness (from grade 0 to III according to Yanagihara, with a median of 2). Median grades of all GRBAS features were: G-2, R-1, B-0, A-0.5, S-2. We found no correlation between the grade of hoarseness (derived from the acoustic and perceptual analysis) and VHI scores, but there was a correlation between VHI score and the severity of sensory complaints (*r* = 0.84). Patients reporting throat pain scored higher on the VHI. In the whole study group we found positive correlations between grade of hoarseness according to Yanagihara and G score (*r* = 0.95) as well as S score (*r* = 0.86).

**Table 2:** Results of LVS examination and voice assessment

Patient no	LVS	Grade of hoarseness according to Yanagihara	GRBAS	VHI
1	Glottal hypofunction, hyperfunction of the supraglottis and lower pharynx, periodical paradoxical movements of the vocal folds	II/III	G2R1B2A1S2	81
2	Hyperfunction of the glottis, periodically observed exacerbated laryngeal closure reflex	II/III	G2R0B1A1S2	44
3	Full phonatory and respiratory mobility, periodic slower movement of the right vocal fold, mucosal dryness	0/I	G1R1B0A0S1	30
4	Hyperfunction of the glottis	0	G0R0B0A0S0	64
5	Hyperfunction of the glottis	III	G3R2B0A0S2	50
6	Right-sided laryngeal paralysis	II	G2R1B0A1S2	9

Table 3 shows the patients' electromyographic findings. In nearly all the patients their LSN showed up as a neuropathic EMG recording. Only in patient 1 did the recording meet the criteria for myopathy [26]: her low EMG amplitude was regarded as indicating muscle weakness, and under endoscopic examination the patient showed features of glottal insufficiency. In all other patients, recordings showed neuropathic features. Neuropathic abnormalities were observed in both the muscles innervated by the superior laryngeal nerve (CT) and the recurrent laryngeal nerve (TA). Abnormal high-amplitude neuropathic units were more frequently recorded from the CT muscle than from the TA muscle. It was noted that when the mean amplitudes in CT was above 500 μV, numerous or very numerous high-amplitude units were observed. One of the subjects (patient 4), who complained only of sensory impairment, was characterized by abnormal EMG recording only in the CT muscle (TA recording was normal). In another patient (patient 5), a correspondence was observed between the location of the reported symptoms and the location of the deviations in the EMG recording. Patient 5 localized sensory complaints on the right side, matching abnormalities in the EMG recording, which were only visible in the right CT muscle. At the same time, it was noted that in all patients who reported sensory dysfunction, an abnormal neuropathic EMG recording was present in the CT.

**Table 3:** Electromyographic findings in two muscles from 6 patients with laryngeal sensory neuropathy

Patient	Symptom duration (months)	Side	Cricothyroid muscle				Thyroarythenoid muscle			
			Phonation			Rest	Phonation			Rest
			Mean amplitude (μV)	Maximum amplitude (μV)	Large MUPs		Mean amplitude (μV)	Maximum amplitude (μV)	Large MUPs	
1	16	Right	205	252	–	Fibryl	118	128	–	–
		Left	210	246	–	Fibryl	140	174	–	–
2	10	Right	109	124	–	–	181	656	–	–
		Left	335	1957	+ + +	Residual act	167	331	+ +	Residual act
3	8	Right	131	273	–	–	273	2446	+ + +	Residual act
		Left	169	585	+ +	Residual act	232	687	–	–
4	7	Right	158	760	+	Fibryl	179	974	–	Residual act
		Left	184	824	+	Fibryl	170	900	–	–
5	8	Right	172	679	+ +	Residual act	187		+	Residual act
		Left	183	456	–	Fasciculations	149		–	–
6	8	Right	148	300	–	–	137	336	–	–
		Left	193	426	+	Residual act	185	898	+ +	Residual act

When the maximum amplitude in the cricothyroid was > 500 μV, multiple high-amplitude motor unit potentials (MUPs) were always observed. [act = activity]

**Fig. 1:**
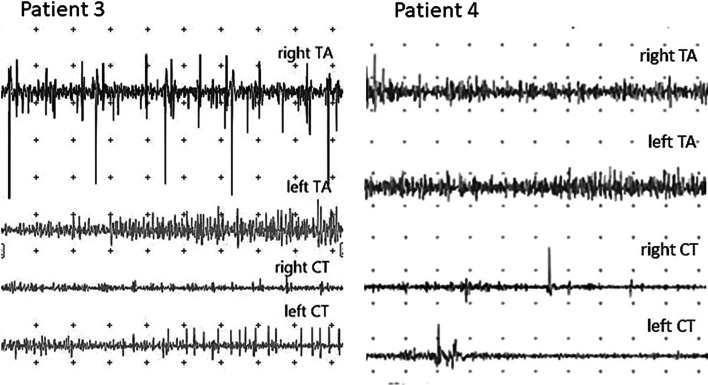
Electromyographic recordings of two patients with LSN. At left are recordings from patient 3 who exhibited multiple neuropathic lesions from the CT and TA muscles. At right are recordings from patient 4 who had neuropathic lesions detectable only from the CT muscles 200 microvolt gain, 40ms per division sweep speed

Figure 1 shows examples of electromyographic recordings from two patients with LSN.

Patients with LSN symptoms were statistically analyzed by comparing the severity of EMG changes with the severity of hoarseness (from perceptual voice analysis) as well as VHI score. The intensity of EMG changes in the CT (expressed as the number of neuropathic units) correlated moderately with the severity of dysphonia (*r* = 0.46). By way of contrast, for the TA muscle we found no correlation between the severity of hoarseness and the amount of neuropathic deviation on EMG examination. Moreover, we did not observe a correlation with VHI score.

Patients showed abnormal activity of the CT muscle not only during phonation but also during respiratory phases. From examination of the TA muscle, neuropathic abnormalities were registered in 4 patients, suggesting concomitant motor nerve fiber damage in addition to sensory nerve damage. Of the first 5 patients, 3 displayed abnormal neurogenic units during phonation in the TA muscle (2 unilateral, 1 bilateral). Only patient 3 showed endoscopic features of nerve paresis (this patient also had the most intense EMG changes in the TA).

The results of patient 6 (with long-standing right-side laryngeal paralysis), suggest there may be additional damage to the laryngeal innervation on the left side due to complaints that occurred after COVID-19 infection but which were not present before. The analysis of the results did not show a correlation between the severity of hoarseness and the severity of neuropathic changes in the TA muscle. An interesting observation is the presence of increased tension of the examined muscles at rest in patients in whom neuropathic activity was observed during volitional activity.

## Discussion

This paper has presented observations of patients with sensory neuropathy of the larynx following COVID-19 infection. Due to epidemiological strictures, ENT diagnostics was curtailed during the infectious phase so the LSN problem seems to have been underestimated. Of the 6 patients seeking our help for complaints due to LSN, about half were professional voice users. Helding has pointed out that LSN seems to strongly affect the singing population [19]. Despite the lack of a comprehensive literature, perhaps due to under-reporting, LSN is mentioned in a review of the COVID-19 literature as a potential complication [27]. In addition to post-viral LSN, Orsucci lists three other chronic medical conditions affecting the larynx which can be related to COVID-19 infection [14]. Those medical complications, affecting vocal production, are: intubation and cough-related injury, post-viral vocal fold paralysis or paresis, and chronic vocal fatigue [27]. The true incidence and prevalence of post-COVID-19 LSN is unknown [19].

The most commonly reported complaints in people with post-COVID LSN were periodic hoarseness of varying severity, discomfort or even pain in the pharynx and larynx, and vocal fatigue. A possible mechanism behind the symptoms is damage to the sensory nerves, leading to axonal degeneration and synkinesis. During regeneration and healing, axons from one sensory receptor may connect to fibers that previously carried signals from a different sensory receptor [28]. As Latremoliere points out, this damage commonly starts in unmyelinated fibers of afferent nerves [28]. Other authors also suggest a role for central sensitisation of the afferent reflex [29]. In the material of our work we found paradoxical vocal fold movements in 2 patients, and in one other the laryngeal closure reflex was also disturbed.

A comparison of the VHI questionnaire results with exacerbation of hoarseness as rated by acoustic voice analysis showed no correlation. The main complaint of LSN patients was laryngeal discomfort. Its severity (pain in the laryngeal region) correlated strongly with the handicap index. Sensory signals arising from the laryngeal mucosa are transmitted mainly by the internal branch of the superior laryngeal nerve in addition to branches of the recurrent laryngeal nerve. In our research, we recorded responses coming from the motoneurons of both these nerves. As reported in previous studies of sensory neuropathy, nerve fiber damage can affect both sensory and motor fibers.

We found that an increase in the severity of LEMG abnormalities in the CT correlated with the severity of dysphonia (as assessed from the spectrogram) and with associated motor dysfunction. In a study by Norris on 12 patients with symptoms of LSN, 75% exhibited evidence of motor neuropathy on laryngoscopy [6]. In our study 5 of 6 patients with post COVID-19 LSN had neuropathic changes in the superior laryngeal nerve (which is the main sensory nerve of the larynx) and of the recurrent laryngeal nerve (the main motor nerve of the larynx), but only 2 of patients had features of periodical motor dysfunction visible in LVS.

According to Daia, EMG studies can reveal demyelinating polyneuropathy changes in patients over the course of COVID-19 infection and recovery [30]. According to the author, elements of myopathy could be a new pathological entity in COVID-19 [30]. Myopathic changes in the EMG are observed in severe demyelinating neuropathy and suggest a direct action of COVID-19 on muscular fibers [30]. In this study, we observed myopathic changes in only one patient; this subject had the most severe form of the disease, and her complaints lasted the longest.

Previous publications report treatment of LSN with amitriptyline, gabapentin, and pregabalin [1]. As stated by Norris, patients with evidence of motor neuropathy appear to have better outcomes with neuromodulator therapy [6]. The addition of reflux precautions and acid suppression therapy to neuromodulator therapy is helpful in cases of chronic and recurrent laryngospasm [6]. For the patients in our study, we additionally recommended vitamin B preparations and physiotherapy. So far, we have observed resolution of symptoms with normalization of the EMG recording in just one of the patients (patient 3). Laryngeal sensory neuropathy is a diagnosis of exclusion. There is no defined way to test internal branch of the superior laryngeal nerve or sensory branches of the recurrent laryngeal nerve. The study indirectly searched for abnormalities in the motor innervation of the larynx on the basis of the LEMG results. Neuropathy of sensory fibers does not mean that there is an obligatory motor fibre neuropathy and vice versa, but literature data show a very high rate of co-occurrence of the disfunctions in post-viral lesions. The abnormal LEMG findings presented in this study point to damage of the laryngeal innervation originating from the superior laryngeal nerve. Abnormal activity from the CT muscle may indicate the co-occurrence of sensory neuropathy. In the authors opinion this diagnosis permits differentiation from laryngeal hypersensitivity which is symptomatic of other diseases [4, 7, 31]. The detection of SLN injury on LEMG examination permits the diagnosis to be documented and the severity of the condition to be assessed. On this basis, treatment can be modified with elements of neurological therapy. In addition, the awareness of long-term nerve regeneration prepares the patient for the treatment process.

## Conclusions

Sensory neuropathy of the larynx may be one of the long-lasting complications of SARS-COV-2 infection.

Laryngeal sensory neuropathy is characterized by a variety of symptoms, of which the most common is dysphonia of various degrees.

Voice professionals are more likely to be those seeking medical attention for symptoms of post-COVID-19 LSN.

To diagnose LSN, it is helpful to use electromyography of the muscles innervated by the superior laryngeal nerve and the inferior laryngeal nerve (CT and TA respectively).

The degree of neuropathic changes seen by EMG of the CT muscle broadly corresponds to the severity of dysphonia.

## Data Availability

Data are available on request.
